# Quality Control Measures over 30 Years in a Multicenter Clinical Study: Results from the Diabetes Control and Complications Trial / Epidemiology of Diabetes Interventions and Complications (DCCT/EDIC) Study

**DOI:** 10.1371/journal.pone.0141286

**Published:** 2015-11-03

**Authors:** Gayle M. Lorenzi, Barbara H. Braffett, Valerie L. Arends, Ronald P. Danis, Lisa Diminick, Kandace A. Klumpp, Anthony D. Morrison, Elsayed Z. Soliman, Michael W. Steffes, Patricia A. Cleary, the DCCT/EDIC Research Group

**Affiliations:** 1 Department of Medicine, University of California San Diego, La Jolla, California, United States of America; 2 The Biostatistics Center, George Washington University, Rockville, Maryland, United States of America; 3 Department of Laboratory Medicine and Pathology, University of Minnesota, Minneapolis, Minnesota, United States of America; 4 Department of Ophthalmology and Visual Sciences, University of Wisconsin, Madison, Wisconsin, United States of America; 5 Department of Medicine, University of South Florida, Tampa, Florida, United States of America; 6 Department of Epidemiology and Prevention, Wake Forest School of Medicine, Winston Salem, North Carolina, United States of America; Swinburne University of Technology, AUSTRALIA

## Abstract

Implementation of multicenter and/or longitudinal studies requires an effective quality assurance program to identify trends, data inconsistencies and process variability of results over time. The Diabetes Control and Complications Trial (DCCT) and the follow-up Epidemiology of Diabetes Interventions and Complications (EDIC) study represent over 30 years of data collection among a cohort of participants across 27 clinical centers. The quality assurance plan is overseen by the Data Coordinating Center and is implemented across the clinical centers and central reading units. Each central unit incorporates specific DCCT/EDIC quality monitoring activities into their routine quality assurance plan. The results are reviewed by a data quality assurance committee whose function is to identify variances in quality that may impact study results from the central units as well as within and across clinical centers, and to recommend implementation of corrective procedures when necessary. Over the 30-year period, changes to the methods, equipment, or clinical procedures have been required to keep procedures current and ensure continued collection of scientifically valid and clinically relevant results. Pilot testing to compare historic processes with contemporary alternatives is performed and comparability is validated prior to incorporation of new procedures into the study. Details of the quality assurance plan across and within the clinical and central reading units are described, and quality outcomes for core measures analyzed by the central reading units (e.g. biochemical samples, fundus photographs, ECGs) are presented.

## Introduction

Ensuring high-quality data collection is essential for performing reliable analyses to achieve meaningful conclusions from the study data. Several factors can impact data quality and completeness, including the accuracy of instruments and measurement techniques, the standardization of data collection across clinical centers, and consistency of analysis in the central reading centers [1–3]. Longitudinal cohort studies face additional challenges that include advances in technology, changes in the availability of materials and equipment, and staff turnover. Therefore, robust quality assurance procedures must be developed and implemented to minimize potential sources of error during and after data collection.

Several longitudinal studies have described their quality assurance procedures [4–7]. However, reports on quality assurance practices in large multicenter longitudinal studies, especially ones that have spanned decades are limited. The Diabetes Control and Complications Trial (DCCT: 1983–1993) was a multicenter, randomized 10-year clinical trial designed to compare the effects of intensive vs. conventional diabetes therapy on the development and progression of microvascular and neuropathic complications in 1,441 participants with type 1 diabetes mellitus. The DCCT demonstrated that intensive glycemic control profoundly reduced the early manifestations of microvascular and neuropathic complications [8]. The Epidemiology of Diabetes Interventions and Complications (EDIC: 1994-present) study was initiated as an observational follow-up of the DCCT cohort to examine the longer-term effects of the DCCT assigned therapies on microvascular, macrovascular and neuropathic complications [9]. EDIC is currently in its 22^nd^ year of data collection and is following 94% of the surviving cohort. The primary aim of this report is to provide an overview of the DCCT/EDIC quality assurance processes employed over the past 30 years to maintain consistent high-quality data collection.

## Methods

### Structure of the DCCT/EDIC Study

The DCCT was comprised of 29 clinical centers located in the United States (US) and Canada, three central reading units and a coordinating center. The clinical centers were responsible for recruitment, enrollment and protocol implementation activities. Prospective subjects with type 1 diabetes between the ages of 13–39 years with minimal or no microvascular complications and no macrovascular disease were evaluated during an extensive 4 month screening process. Eligible subjects (n = 1,441) were randomly assigned to either intensive or conventional therapy. Intensive therapy was designed to achieve near normal glycemia using 3 or more daily injections of insulin or an insulin pump, with dosing decisions based on frequent daily self-monitoring of blood glucose. Conventional therapy focused on the absence of hypoglycemia and hyperglycemia and was consistent with current standard clinical care practices. Staff members at the clinical centers were responsible for ongoing medical care and education, and completion of protocol-mandated evaluations. All biochemical analyses were performed by the Central Biochemistry Laboratory (CBL: University of Minnesota, Minneapolis MN); stereoscopic fundus photographs were graded by the Central Ophthalmologic Reading Center (CORU: University of Wisconsin, Madison WI); and electrocardiograms (ECGs) were read by the Central ECG Reading Center (CERC: University of Minnesota, Minneapolis MN). Data collection forms were sent from the clinical centers to the Data Coordinating Center (DCC: George Washington Biostatistics Center, Rockville MD) to be entered into the central database, and data files from the central units were transmitted and merged into each participant’s centralized data file. The DCC provided the overarching organization, coordination and support structure for the study. Ongoing data review based on pre-defined parameters specified in the protocol identified existing safety concerns and the DCC monitored treatment implementation and medical outcomes.

**Fig 1:**
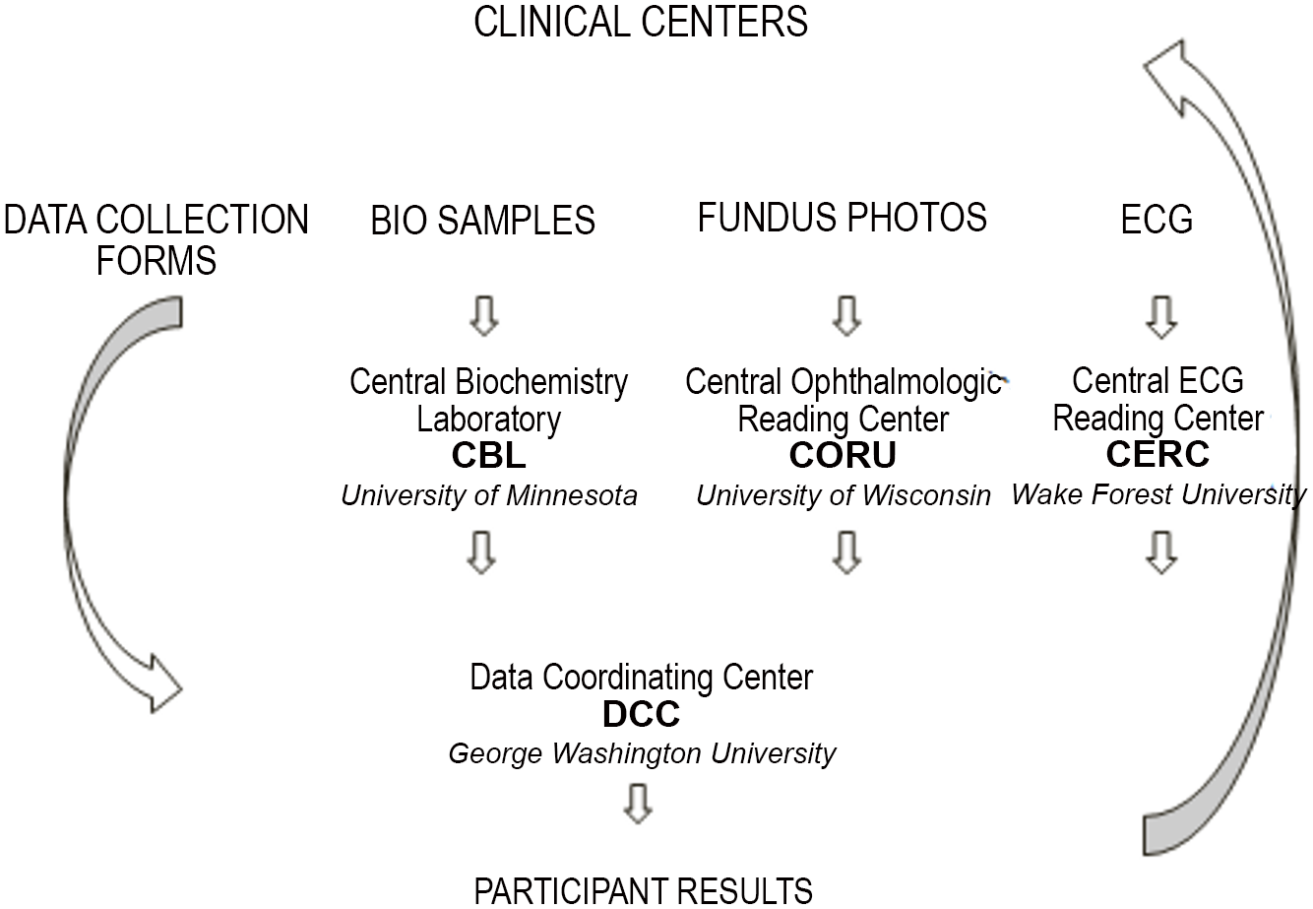
Data Flow in the DCCT/EDIC Study. The CERC was located at the University of Minnesota, Minneapolis, MN between 1983–2004, and at Wake Forest University, Winston Salem, NC from 2005-present.

Following the DCCT, the participants were invited to enroll in the EDIC longitudinal observational follow-up study. Two central coordinating centers, each with distinct functions, support and oversee all of the operational aspects of the EDIC study. The clinical centers are responsible for direct interface with the study participants and data collection, and the central reading units provide technical expertise for the evaluation of laboratory samples, fundus photographs and ECGs (Fig 1). Institutional Review Board (IRB) approval was obtained by each of the individual clinical centers as well as the Data Coordinating Center. Written informed consent was provided by each of the study participants at the clinical centers. Consent for minor participants at study screening and randomization was provided by the minor’s parent or legal guardian, as directed by local institutional guidelines.

### Coordinating Centers

#### Data Coordinating Center

Data management and analytic support for the EDIC study are provided by the Data Coordinating Center (DCC: George Washington University Biostatistics Center, Rockville MD). The DCC coordinates the development of data collection instruments, designs and maintains a centralized data management system and a secure internal study website, coordinates the transmission of data from the central reading units, distributes concise reports of participant results, and develops periodic monitoring reports for oversight committees and funding agencies. Statisticians at the DCC are responsible for developing analytic plans, performing analyses for all core data and ancillary studies, and supporting the development of scientific publications and presentations.

#### Clinical Coordinating Center

Overall coordination and fiscal management of the clinical centers is provided by the Clinical Coordinating Center (CCC: Case Western Reserve, Cleveland, OH). The CCC supports the clinical aspects of protocol implementation and oversees staff transitions at the clinical centers. With the addition of ancillary protocols involving new measurements, the CCC works closely with investigators to standardize equipment and processes across the clinical centers. Fiscal responsibilities include maintenance of subcontracts and budget management oversight at the clinical centers. Through its activities with the clinical centers, the CCC supports the quality assurance efforts that are overseen by the DCC.

### Clinical Centers

Twenty seven of the original 29 clinical centers in the US and Canada participate in the EDIC Study, and participants from the 2 remaining DCCT clinics continue to participate and are being followed at a nearby EDIC clinical center. Study personnel at each center include the principal investigator and study coordinator, with additional administrative, research and/or nursing support based on participant enrollment and local clinical center resources and preferences. Standardized processes for the collection of outcome measures are defined in the EDIC Manual of Operations (MOO) and utilized by all clinical centers. The MOO also defines specific training and certification activities that are required prior to collection of study data and provides guidance related to participant communication and study retention [10].

### Central Reading Centers

Central reading centers are responsible for the analysis of biochemical laboratory results, fundus photographs, and ECGs. Additional central reading centers are established to support ancillary studies that require additional or unique expertise beyond that found within the existing reading centers. Each reading center has defined quality control systems in place to monitor and evaluate equipment functioning, validate consistency of measurements over time and across various personnel and systems, and has specified staff training and retraining activities. Changes in equipment or processes that may affect data quality and integrity are evaluated for measurement consistency to ensure validity of longitudinal analyses over time. Additionally, blinded re-reading of a standard set of measures at pre-defined intervals for study procedures, such as fundus photographs or ECGs, and collection of split-duplicate samples for biochemical laboratory samples are used to evaluate and track potential differences in instrument characteristics, personnel behaviors and/or product variations over time. An overview of the various measures, specimen requirements, collection procedures, testing frequency, as well as the quality and precision plan is presented in Table 1.

**Table 1:** Quality Control Monitoring in the DCCT/EDIC Study.

Procedure	Measurement	DCCT Frequency	EDIC Frequency	Specimen Criteria	Centrally Analyzed^a^	Analytic Precision^b^
**PHYSICAL EXAM**						
Blood Pressure	Resting Systolic, Diastolic	Quarterly	Annually	Sitting right arm	NO	NA
	Ankle Brachial Index	Not done	Biannually	Supine bilateral	NO	NA
Height	Height to nearest 0.1 cm	Quarterly	Annually	1.0 cm^c^	NO	NA
Weight	Weight to nearest 0.1 kg	Quarterly	Annually	0.2 kg^d^	NO	NA
Waist, Hip Girth	Girth to nearest 0.5 cm	Not done	Annually	0.5	NO	NA
**BLOOD MEASUREMENTS (non-fasting)**	Glycosylated hemoglobin	Quarterly	Annually	Whole Blood	YES	Split Duplicate
	Creatinine	Annually	Annually	Frozen Serum	YES	Split Duplicate
	Cystatin-C	Not done	Annually	Frozen Serum	YES	Split Duplicate
**BLOOD MEASUREMENTS (fasting)**	Cholesterol	Annually	Even randomization anniversary	Frozen Serum	YES	Split Duplicate
	Triglycerides	Annually	Even randomization anniversary	Frozen Serum	YES	Split Duplicate
	HDL-Cholesterol	Annually	Even randomization anniversary	Frozen Serum	YES	Split Duplicate
	LDL-Cholesterol	Annually	Even randomization anniversary	Frozen Serum	YES	Split Duplicate
**URINE MEASUREMENTS**						
Creatinine Clearance	Urine Creatinine	Annually	Odd randomization anniversary	Frozen Serum and Urine	YES	Split Duplicate
Albumin Excretion	Urine Albumin	Annually	Odd randomization anniversary	Frozen Serum and Urine	YES	Split Duplicate
**FUNDUS PHOTOGRAPHY**	Stereoscopic Fundus Photography	Every 6 months	Every 4 years based on randomization anniversary	7-Standard Fields	YES	Reread
**ELECTROCARDIOGRAM**	Electrocardiogram	Annually	Annually	12-Lead Resting	YES	Reread

^a^ Data analyzed at the central reading units (e.g. CBL, CORU, CERC);

^b^ EDIC-specific quality assessments

^c^ Height: two measurements within 1 cm; if not, measure twice more

^d^ Weight: two measurements within 0.2 kg; if not, measure twice more

## Data Quality Assurance Plan

The EDIC data quality assurance plan relies on the utilization of procedures and processes that are standardized, reproducible, and measureable over time. Each central reading unit must ensure uninterrupted, reproducible and comparable data accuracy and reliability over time and especially whenever equipment or procedural changes are necessary. The individual quality assurance plans at the DCC, clinical centers, and central reading centers are described.

### Data Coordinating Center

The DCC is instrumental in establishing procedures for data quality and adherence and in developing study monitoring reports for internal and external evaluation. In 2012, the DCC transitioned from a paper-based legacy system to a custom built web-based data acquisition and management system called MIDAS (Multi-modal Integrated Data Acquisition System), a proprietary system developed by the George Washington Biostatistics Center and used by all clinical research studies coordinated there. Many aspects of the DCC’s current quality assurance program are integral parts of the MIDAS system, beginning with the development, testing and implementation of data collection forms. All content for data collection forms and instruments are developed by a sub-committee of study group members and reviewed and finalized successively by the Data Quality Assurance, Adherence Monitoring, and Executive Committees. Once approved, the forms are implemented through three sequential databases in MIDAS: one for form development, one for testing data collection forms, and a third for production where real data are collected.

MIDAS provides on-screen, real-time data validation. Data entry personnel at the clinical centers are immediately notified while entering a data item if it falls outside a valid range, violates some other criterion (e.g. skip pattern), or when a required data item is omitted. All data are checked for discrepancies (within or across forms) nightly and reports are generated in MIDAS frequently. Reports are sent to the clinical centers to correct or to explain why the data values are missing or invalid. Quality control includes discrepancy management through bi-weekly DCC staff meetings to review clinical center responses to data issues. MIDAS maintains an audit trail of all data modifications throughout the lifetime of the study.

Data security is also incorporated directly into the MIDAS system. Authorized personnel must be registered and are assigned a level of access based on their responsibilities. All data entered into the MIDAS system are backed up on a daily basis to ensure data integrity. The DCC performs a yearly database lock which includes additional data validation and verification prior to reporting results annually to the EDIC Observational Study Monitoring Board. The DCC’s data quality assurance plan complies with the standards, policies, and procedures of the Health Insurance Portability and Accountability Act (HIPAA).

### Clinical Centers

#### Certification Procedures

Individuals performing the procedures to acquire the multiple outcome measurements are trained, tested, and certified as competent prior to performance of measurements or collection of data involving study participants. Periodic retraining and certification reinforce consistency and standardize the training of new personnel. Once certified, the individual is assigned a unique identification number which is tracked to ensure that certified individuals are collecting the appropriate data (Table 2). Additional training and certification activities are performed prior to implementation of new measurements or approved ancillary studies to ensure uniformity of measurements and data collection across the clinical centers. Re-certification is recommended for individuals not involved in the study for 3 or more years prior to performing any data collection activities.

**Table 2:** Certification and Standardized Processes in the DCCT/EDIC Study.

Procedure	Certification requirements	Standardized methods	Standardized equipment/ materials
Medical history / physical exam	Completion of data collection form on 2non-study participants	X	X
Blood pressure	Knowledge of study-specific measurement criteria	X	X
Data entry	Training module; entry and submission of “test” data form	X	X
Shipment of biosamples	Shipment of cold and frozen “test” samples	X	X
Visual acuity	Use of study-specific visual acuity techniques; training by local ophthalmology staff	X	X
Ophthalmology exam	Submission of curriculum vitae that documents ophthalmology expertise; knowledge of exam requirements and form completion	X	
Electrocardiogram	Paper submission of 2 “test” subject ECG’s to CERC; successful digital transmission of “test” ECG	X	X

#### History and Physical

A standardized history form is used to document specific participant self-reported outcomes. Designated outcomes require the completion of event-specific verification forms based on additional medical information obtained from the participant’s health care providers and/or local healthcare institutions. External medical records documenting all cardiovascular events and participant deaths are reviewed and adjudicated by the Morbidity and Mortality Committee, which is comprised of select principal investigators representing the clinical centers and the ECG central reading unit.

#### Blood Pressure Measurements

Blood pressure is measured using standardized procedures and equipment at the clinical centers as outlined in the EDIC MOO. Two sitting measurements, taken on the right arm unless contraindicated, are obtained at each participant’s visit, following 5 minutes at rest and again 2 minutes later. Additionally, dual Doppler blood pressure measures are performed and the average systolic pressure recorded for bilateral dorsalis pedis, posterior tibial and brachial are used to compute ankle:brachial index. Every four months, study-wide blood pressure data is reviewed by the Data Quality Assurance (DQA) committee to assess digit preference, frequency of missing data, as well as measurement means, ranges, reliability, variability, and frequency of extreme values. Problems and/or trend identification guide re-training efforts.

### Central Reading Centers

#### Central Biochemistry Laboratory

Biospecimen collection, processing and shipment across clinical centers are standardized via protocols defined in the EDIC MOO. Biospecimen testing is performed in the Clinical Laboratory Improvement Amendments (CLIA)-certified and College of American Pathologists (CAP)-inspected CBL. In accordance with CLIA and CAP, the CBL performs daily quality control testing and participates in external CAP proficiency survey testing for all analytes. External reference materials are tested and monitoring programs are followed; these include the Centers for Disease Control (CDC) lipid comparability program, testing of National Institute of Standards and Technology (NIST) reference materials for glucose and creatinine measurements, and the National Glycohemoglobin Standardization Program (NGSP) and International Federation of Clinical Chemistry (IFCC) monitoring of glycated hemoglobin. Split duplicate samples collected by the clinical centers are analyzed and the results are compared; these data are reviewed quarterly by the DQA committee (Table 3).

**Table 3:** Methods in the DCCT/EDIC Study.

Procedure	Measurement	Method or Assay	Dates of Use
**PHYSICAL EXAM**			
Blood Pressure	Resting Systolic, Diastolic	Sitting, right arm reading with sphygmomanometer; average of 2 measurements	1983-Current
	Ankle Brachial Index	Average of 2 measurements at each of 6 locations	1994-Current
Height	Height to nearest 0.1 cm	2 measurements within 1 cm; if not, measure twice more	1983-Current
Weight	Weight to nearest 0.1 kg	2 measurements within 0.2 kg; if not, measure twice more	1983-Current
Waist, Hip Girth	Girth to nearest 0.5 cm	2 measurements within 0.5 cm; if not, measure twice more	1994-Current
**BLOOD MEASUREMENTS (non-fasting)**	Glycosylated hemoglobin	High-performance ion-exchange liquid chromatography	1983-Current
	Serum Creatinine	Jaffe rate	1983-May 2007
	Serum Creatinine	Enzymatic method	June 2007-Current
	Cystatin-C	Rabbit monospecific anti-human Cystatin-C antiserum immunoassay	August 2003-October 2012
**BLOOD MEASUREMENTS (fasting)**	Cholesterol	Cholesterol oxidase spectrophotometry	1983-Current
	Triglycerides	Glycerol-blanked glycerol kinase/glycerol oxidase spectrophotometric	1983-Current
	HDL-Cholesterol	Magnesium dextran precipitation and cholesterol oxidase spectrophotometric	1983-Current
	LDL-Cholesterol	Calculated, Friedewald equation	1983-Current
**URINE MEASUREMENTS**			
Creatinine Clearance	Urine Creatinine, 4-hour	Jaffe rate	1983-May 2007
	Urine Creatinine, 4-hour	Enzymatic	June 2007-July 2012
	Urine Creatinine, random	Enzymatic	August 2012-Current
Albumin Excretion	Urine Albumin, 4-hour	Fluorescent immunoassay	1983-July 2012
	Urine Albumin, random	Immunoturbidimetric	August 2012-Current
Albumin Excretion Rate		Calculated	1983-July 2012
Albumin-Creatinine Ratio		Calculated	August 2012-Current
**FUNDUS PHOTOGRAPHY**	Stereoscopic Fundus Photography	Final ETDRS Grading Scale for Retinopathy and Macular Edema [11]	1983-Current
**ELECTROCARDIOGRAM**	Electrocardiogram	Revised Minnesota Code [12]	1983-Current

The quality control plan evaluates the sample management process, from time of collection, processing and storage at the clinical center, to shipment and eventual sample measurement at the CBL. Deviations are identified and efforts made to determine the location and cause of the deviation. This involves re-analysis of the samples in question, communication with the clinical center about discrepancies related to sample processing, labeling or shipment, and/or tracking of the results from the CBL to the DCC. Causes of all deviations are documented and communicated to the clinical centers in an effort to reduce recurrence.

#### Central Ophthalmologic Reading Center

Bilateral stereoscopic color fundus photographs were obtained from study participants at baseline and semi-annually through the DCCT, and during EDIC at baseline and every 4 years based on each participant’s randomization anniversary, plus full cohort photos during EDIC years 4 and 10. A standardized photographic protocol was modified from the 7-Standard Fields protocol of the Early Treatment Diabetic Retinopathy Study (ETDRS) [13] and clinical sites and equipment were certified by the ophthalmologic reading center. Until 2011, film images were obtained as 35mm color slides. Since then, digital images of the fundus have been obtained and graded by the CORU using methods adapted from the evaluation of film. Each set of images is given a quality score by reading center technicians. Submissions of only fair or poor quality have the underlying reason for suboptimal quality identified, whether due to technical quality (focus, illumination, field definition, etc.) or eye-related (cataract, small pupil, vitreous hemorrhage, etc.). Image quality is compiled for each clinical site; sites with recurrent inadequate performance are counselled by reading center photographers until quality meets acceptable levels.

Fundus photographs are graded by technicians who are trained and certified by the reading center using light boxes with stereoscopic viewers for film and a modified display and analysis application (IMAGEnet: Topcon Medical Systems, Paramus NJ) with a hand held stereoscopic viewer for digital images. Diabetic retinopathy is assessed in each field according to the presence and severity of index lesions (including microaneurysms, hemorrhages, hard exudate, retinal thickening, venous beading, intraretinal microvascular abnormalities, and neovascularization) according to ETDRS procedures [13]. The evaluation consists of two independent gradings for each eye, after which a processor is run that flags eyes with discrepant evaluations. The grading discrepancies are adjudicated by a third grader. If the grading did not require adjudication, the first grade is considered the grade of record. If adjudication is indicated, the third grading becomes the grade of record. Eyes that are considered ungradable by only one of the first two graders are reviewed.

Greater efficiency in quality evaluation was realized through a modification of the earlier work flow, where every submission was given a technical quality score by a specialized imaging technician. A process was developed whereby the grader evaluates the quality of the image set. While the image quality of the vast majority of the submissions is adequate, only those image sets with suboptimal image quality are given a detailed technical score and receive a more detailed technical quality evaluation.

Grading reproducibility is monitored through re-grading of submissions identified by the DCC. ETDRS retinopathy severity level and other ordinal variables are analyzed using a weighted Kappa and percentage agreement while continuous variables, such as area of macular thickening, are analyzed by intra-class correlation, scatter plots and agreement within a specified limit and reported to the DQA committee [14]. The range of the weighted Kappa statistic for DR severity by eye was 0.76–0.81 for the last 3 annual exercises, which is consistent with historical reproducibility [15].

#### Central ECG Reading Center

Quality control (QC) measures were instituted during the DCCT and applied unchanged in the EDIC study. Senior staff continuously monitor ECG quality and identify any procedural errors in ECG acquisition. Lead reversal is particularly problematic and more common than is generally recognized for the standard resting ECG. Quality grades assigned to each ECG are used to compile continuous quality trend analysis data to identify emerging problems, which is particularly important as ECG personnel change over the duration of study.

A series of quality control (QC) reports are sent regularly to the DCC. The ECG quality grades are 1, 3, and 5. Grade1 is perfect while grade 3 reflects an increasing amount of artifacts. ECGs with grade 1 and grade 3 are fully retrievable for study data, whereas grade 5 ECGs (the poorest quality grade) are generally not suitable for data analysis. Some quality grade 5 ECGs can be still usable for classification, but are limited particularly with classification of borderline abnormalities. The overall goal for each clinical center is to ensure the fraction of poor quality recordings (grade 5) remains below 5% of the total submitted from the clinical center throughout the study. Declining quality, by individual technicians, clinics or the study as a whole, are triggers for site investigation and retraining.

Following training and certification of the ECG coders, a quarterly monitoring for inter-and intra-coders variability is installed. Test library sets of ECGs which have previously been carefully coded and verified by senior electrocardiographers are used for blind testing of the visual coding process. The re-coded data are tabulated no less than quarterly and contingency tables indicating the frequency of deviations from the standard (off-diagonal elements) are generated. A Kappa statistic is determined to evaluate coding consistency [14]. Any systematic trends and/or significant deviations from the standard trigger appropriate corrective action and retraining of the coding personnel. In addition, a 1% coding daily audit is performed by the Coding Supervisor. Significant changes in Kappa statistics are carefully examined for the source of coding change, and to determine if there is a decrement in rule application or change in coding staff.

To provide repeatability of coding, all EDIC study ECGs are coded by the same coder. All coded ECGs are reviewed by another coder before being sent for data entry. For the EDIC study, the codes are checked once again at the time of clinical review reporting, and once again (fourth time) at the time of monthly reporting to the DCC.

Calibration of machines for a standard gain of 10 mm = 1 mV electrocardiographs are set invariably. During the reading process, the ECG coders check the calibration of the ECG machines. Frequent background in specific leads in specific clinics flags machine error especially if there have been no staff changes in that clinic that may account for the change in quality.

## Role of the Data Quality Assurance Committee

Maintenance of data quality and integrity from collection through dissemination of the results is the collective responsibility of the entire study group. The DQA committee, comprised of representatives selected by the Executive committee from the clinical centers, central reading units and DCC, is responsible for quality oversight throughout the study. The DQA committee convenes via teleconference every four months to review study-wide data quality and data trends over time. The DQA committee evaluates additional monitoring measures for ancillary studies or new study procedures, and may recommend the implementation of additional quality control measures to ensure data accuracy and reliability at the clinical centers and/or central reading units.

Split-duplicate samples are used to monitor reliability and reproducibility of biochemical laboratory specimens within a defined time period and over time. Mean within specimen coefficient of variation (CV) is the average of the CVs for the sample pairs. The coefficient of reliability (CR) is an estimate of the proportion of the total variability between individual subject values that is due to differences between the actual values and thus not due to measurement error. The cumulative CV and CR for biochemical measures since the inception of the EDIC study in 1994 are presented in Table 4.

**Table 4:** Quality Results during the EDIC Study (1994–2014).

**BIOCHEMICAL SAMPLES**		**Variability (Coefficient of Variation)**		**Reliability (Coefficient of Reliability)**	
**Serum**					
Glycosylated hemoglobin		0.48		0.998	
Creatinine		2.14 / 1.28		0.982 / 0.998	
Cholesterol		1.3		0.998	
Triglycerides		2.3		0.981	
HDL-Cholesterol		1.8		0.998	
LDL-Cholesterol		2.1		0.989	
**Urine**					
Creatinine^a^ ^,^ ^b^		3.0 / 2.69		0.969 / 0.99	
Albumin^a^ ^,^ ^b^		10.2 / 5.18		0.972 / 0.999	
Albumin Excretion Rate^a^		13.5		0.963	
Albumin-Creatinine Ratio^b^ ^,^ ^c^		8.91 / 6.24		0.997 / 1.000	
Creatinine Clearance^a^		4.3		0.937	
**FUNDUS PHOTOGRAPHY**					
**Image Quality (%)**		**Excellent**	**Fair**	**Borderline**	**Unreadable**
April 1994–August 2008^d^	(n = 12,724)	58	34	8	1
		**High**	**Adequate**		**Inadequate**
September 2008–December 2014^e^	(n = 3,016)	82	16		2
**Diabetic Retinopathy Severity Reproducibility** ^f^ **(%)**		**Weighted kappa (95% CI)**	**Exact agreement**	**Agreement within 1 level**	**Agreement within 2 levels**
Temporal Drift 2013	(n = 156)	0.8 (0.74, 0.86)	66	91.7	94.9
**ELECTROCARDIOGRAM (%)**		**No quality issues**	**Minor technical, readable**		**Unreadable**
January 1994–December 2014	(n = 25,761)	93	6		1

^a^ 4-hour timed urine collections (April 1994 –August 2012)

^b^ Single random urine collections (August 2012 –December 2014)

^c^ Single random urine collections (August 2004 –April 2013)

^d^ During the DCCT through 2008 in EDIC, fundus photo quality was assessed using a 4-tier grading system.

^e^ In 2008, the photo image quality control program expanded to include confidence scoring that allowed the graders to indicate their confidence in the grading of ocular disease as impacted by the quality of the photo set. High—no significant quality issue; Adequate—suboptimal quality interferes with grading; Inadequate—quality that prevents determination of major disease parameters. (CORU internal white paper: Image Confidence Scores, effective 04 Feb 2008).

^f^ Annual re-read of 100 photo pairs across the spectrum of retinopathy severity performed by the CORU to evaluate reproducibility of scoring over time. 2013 Kappa scores consistent with previous photo re-reads.

Annual re-reads of a pre-defined set of ECGs and fundus photographs assess reproducibility of results, presence of inter- and intra-reader variation, and/or potential drift in results over time. Additionally, changes in technical quality of specific measures are identified and clinical centers are notified to review deviations and investigate potential causes. This feedback mechanism facilitates timely problem identification and implementation of quality improvement strategies, which together, promote long-term quality improvement (Table 4). Ancillary procedures are tracked and assessed similarly.

## Management of Process Change

Advances in technology and changing availability of materials have necessitated changes in the collection and analysis processes and/or equipment over time in the DCCT/EDIC. Transition processes are developed to validate the comparability of the results using existing and proposed technology to ensure reproducibility of results. These processes are described below.

### Implementation of Web-based Data Entry

Between 1983 and 2011, the DCCT/EDIC legacy data management system required centralized data entry by trained and certified keyers at the DCC. With implementation of the MIDAS data management system, the burden of data entry was transferred from the DCC to each clinical center. Fifty-seven staff members at 27 clinical centers have been trained and certified to use the web-based data entry system. Three years after the implementation of MIDAS, the average number of days between the participant’s annual visit and MIDAS data entry fell from an average of 51 days with the legacy system to 11 days (90% within 25 days).

With the legacy system, paper-based edit reports and participant results were generated quarterly and mailed to the sites for correction and verification, resulting in a 2–4 month time lapse from data collection to data reporting. MIDAS web-based data editing and reporting modules allow for edits to be resolved weekly and for participant reports to be available for immediate download. The time required for data entry at the clinical centers has been well balanced by the ease of use, rapidity of data editing and availability of time-sensitive data and reports. Implementation of these changes has resulted in improvements in data quality.

Extensive updates to the secure internal EDIC website also allow for easier access to forms, protocols, and information sharing, thereby enhancing data quality.

### Instrumentation and Assay Changes at the CBL

Over the 30-year history of the study, changes and improvements in laboratory instrumentation and/or reagents have necessitated changes in the measurement of analytes. To ensure accurate and precise laboratory results, new methods and modifications of existing methods (including instrumentation changes) are validated prior to implementation. Method and/or instrument evaluation is performed according to the manufacturer’s guidelines when available. In other instances, guidelines adapted from CAP and the Clinical Laboratory Standards Institute (CLSI) are followed as appropriate. Such evaluation may include accuracy, precision, sensitivity, specificity, and carry-over analyses. In addition, reportable range, analytical measurement range, and reference/therapeutic range may be determined.

Two examples of changes in assay methods involve creatinine and cystatin C. Creatinine measurement using the Jaffe method has been shown to exhibit non-creatinine chromogenic interference in samples from patients with diabetes [16,17]. Studies were performed to evaluate comparability and reproducibility of results using current as well as historic stored serum samples. With comparability established, historic creatinine and creatinine clearance values were recalculated and reported. The current enzymatic method has less potential for such interference. The enzymatic method is calibrated against an IDMS-traceable procedure. Cystatin C measurement was initially performed using a nephelometric method. However, evidence of calibrator drift [18] required a change to an immunoturbidimetric method with calibration traceable to a reference material.

Periodically, procedural changes in the collection methods for biochemical samples are warranted. Recognizing the participant burden of a 4-hour timed urine collection, a pilot study was conducted to compare albumin excretion rates from the standard 4-hour timed collection with a single random void [19] to assess the comparability of the two urine collection procedures prior to the study-wide protocol change. Verification of comparability prior to adoption of the simpler method sustained the continued longitudinal evaluation of renal function over more than 30 years of follow-up.

### Transition from Film to Digital Images

During EDIC, digital fundus camera equipment became more commonplace in EDIC clinical centers, access to film stock and certified film development was becoming problematic due to obsolescence, and the quality and resolution of digital fundus images from newer cameras began to approximate the quality of film images. However, troublesome new artifacts and quality issues were found in digital retina images, particularly with respect to illumination and color contrast that was much more variable with digital images than in film. The CORU tested sets of digital and film images on the same eyes to determine the optimal conditions for grading digital images to compare to film images with similar sensitivity and reproducibility [20]. As a minimum standard, fundus camera equipment was required to have a resolution of 3 megapixels and certification submissions had to have good illumination and tonal balance along with the standard quality attributes carried over from film grading. In addition, a post hoc standardized illumination and tonal balance optimization process was developed [21] and applied to all images through a custom software application developed with Dr. Nicola Ferrier, Professor of Computer Sciences, University of Wisconsin-Madison. Through these processes, the reproducibility and agreement of grading between digital and film images of the same eyes became equivalent to historical CORU reproducibility from resampled film image grading.

A pilot study was conducted to compare digital images to film from EDIC clinical centers. Once quality and comparability were verified, clinical center photographers and equipment were certified for obtaining digital images. Procedures were modified for digital photography, however, the same 7 stereoscopic field protocol is used. Quality assessments include artifacts unique to digital imaging. The reading center grading procedures have adapted for the new digital work flow and analysis environment, and graders are allowed limited use of digital tools such as zoom and viewing in the green RGB channel.

### Transition to Digital ECG Recording and Electronic Transmission

The EDIC study implemented digital ECG recording using GE MAC 1200 ECG machines (GE, Milwaukee WI) in late 2014. Unlike non-digital (paper) ECG data collection, the recorded digital signal can be stored indefinitely without the risk of deterioration of the ECG tracings and has the ability to automatically record hundreds of ECG waveform measurements that are logistically impossible to obtain from paper ECGs. The recorded signal is transmitted directly to the ECG reading center via phone lines which speeds up the reading process with less effort and cost. All technicians were trained on how to use the new digital ECG machines, and how to electronically transmit the recorded ECGs to the ECG Reading center.

## Discussion

Data integrity, reliability and reproducibility are important requirements for any research project. In the context of multicenter and/or longitudinal studies, an effective monitoring program that is designed to identify trends, data inconsistencies and process variability and be responsive to temporal technologic changes is essential to ensure validity and comparability of results over time.

The DCCT was designed to identify the relationship between glycemic control and microvascular complications associated with type 1 diabetes. During the DCCT, clinical centers collected an enormous amount of clinical, laboratory, electrophysiologic and behavioral information using standardized protocols, procedures and processes. All laboratory samples were collected and processed by the clinical centers following protocols that defined eligibility criteria for testing, collection sequences and processing instructions. Upon receipt at the CBL, samples were batched and analyzed using standardized processes. Similar collection and analysis activities occurred for fundus photographs, ECG’s and other clinical tests. Standardized data management and defined quality control processes at the DCC enabled identification of data discrepancies, redundancies, and outliers for various clinical evaluations included in the protocol (Table 1).

Quality assurance oversight of the DCCT (1983–1993), and subsequently of the EDIC study (1994—present) has been provided by a named committee (DCCT—Standards and Methods Committee; EDIC—Data Quality Assurance Committee) that includes representation from the clinical centers, central reading units and the DCC.

The Standards and Methods Committee in the DCCT was instrumental in developing standardized clinical methods, reviewing data from the central reading units and providing guidance to the DCC. The committee reviewed quality control parameters, identified inconsistencies, explored sources of variation, and implemented corrective actions as needed to ensure data quality and integrity. In the EDIC study, the Data Quality Assurance Committee continues to provide quality assurance oversight and guide the development of specific quality control processes for new protocol evaluations. Every 4 months, quality measures are reviewed, and parameters such as inter- and intra-reader variability (i.e. fundus photos, CT scans, CMRI), coefficients of reproducibility and reliability of split duplicate laboratory samples, and re-read reproducibility for annual established datasets (photos, ECGs) are assessed. Variations are flagged and the degree of variation is evaluated to determine research and/or clinical significance acutely and over time. Investigations to determine the source of variability are performed by assessing the path from collection at the clinical centers through analysis and reporting at the central units. When indicated, staff re-training is recommended to ensure accuracy and consistency of data collection processes across all of the clinical centers over time.

Transitions to more contemporary methods for data acquisition result in additional time commitments and altered roles and responsibilities at the clinical centers, reading centers and coordinating centers. Additional staff are trained and certification activities are implemented to prevent or reduce risk of loss of data or to minimize any adverse impact on overall data quality. Some transitions improve efficiency and timeliness of data reports, others reduce patient and staff burden during study visits, and yet others ensure the long-term integrity of study data using electronic data capture and storage. In all cases, the procedural changes implemented to date have preserved or improved data completeness and quality.

While data integrity and reliability are essential to achieve meaningful research outcomes, there are practical implications when data results are used to augment the delivery of patient care. DCCT/EDIC participants have access to their clinical research data, currently over 25–30+ years. These results, which are provided to the participant following each annual study visit and, if requested, to their healthcare provider, may be used to monitor health status, track changes in health outcomes over time, evaluate the impact of treatments prescribed by the local healthcare provider and guide clinical decisions that impact the participant’s health. Ongoing use of these results provides early detection and/or treatment of evolving complications and data to monitor disease progression. Ongoing clinical use of these results further underscores the importance of ensuring data quality and comparability of the individual DCCT/EDIC results over time, and contributes to the remarkable and unprecedented high participant retention for over 30 years [22].

## Conclusion

An effective quality control program is well-defined, provides a systematic review of processes, flexibly incorporates additional parameters for new protocol measures, and is supported by a structure that can recognize and adjust to change. The continued success of the DCCT/EDIC study is based on the ongoing collection of high quality data. The DCCT/EDIC quality assurance program provides a framework for early identification of process inconsistencies, collection errors, mechanical variances and personnel differences that may impact the longitudinal validity and comparability of the data results over time. Furthermore, the program described here may serve as a resource for the development of quality assurance programs by other long-term clinical studies.

## References

[1] Whitney CW, Lind BK, Wahl PW. Quality assurance and quality control in longitudinal studies. Epidemiol Rev 1998;20(1):71–80. 9762510 10.1093/oxfordjournals.epirev.a017973

[2] Gassman JJ, Owen WW, Kuntz TE, Martin JP, Amoroso WP. Data quality assurance, monitoring, and reporting. Control Clin Trials 1995 4;16(2 Suppl):104S–136S. 7789140 10.1016/0197-2456(94)00095-k

[3] Neta G, Samet J, Rajaraman P. Quality Control and Good Epidemiological Practice. 2014.

[4] Cushman M, Cornell ES, Howard PR, Bovill EG, Tracy RP. Laboratory methods and quality assurance in the Cardiovascular Health Study. Clin Chem 1995 2;41(2):264–270. 7874780

[5] Bergmann MM, Bussas U, Boeing H. Follow-up procedures in EPIC-Germany—data quality aspects. European Prospective Investigation into Cancer and Nutrition. Ann Nutr Metab 1999;43(4):225–234. 10592371 10.1159/000012789

[6] Hilner JE, McDonald A, Van Horn L, Bragg C, Caan B, Slattery ML, et al Quality control of dietary data collection in the CARDIA study. Control Clin Trials 1992 4;13(2):156–169. 1316830 10.1016/0197-2456(92)90021-q

[7] Prud'homme GJ, Canner PL, Cutler JA. Quality assurance and monitoring in the Hypertension Prevention Trial. Hypertension Prevention Trial Research Group. Control Clin Trials 1989 9;10(3 Suppl):84S–94S. 2680275 10.1016/0197-2456(89)90044-5

[8] The DCCT Research Group. The effect of intensive treatment of diabetes on the development and progression of long-term complications in insulin-dependent diabetes mellitus. N Engl J Med 1993 9 30;329(14):977–986. 8366922 10.1056/NEJM199309303291401

[9] The DCCT/EDIC Research Group. Epidemiology of Diabetes Interventions and Complications (EDIC). Design, implementation, and preliminary results of a long-term follow-up of the Diabetes Control and Complications Trial cohort. Diabetes Care 1999 1;22(1):99–111. 10333910 10.2337/diacare.22.1.99 PMC2745938

[10] The DCCT Research Group. The Diabetes Control and Complications Trial (DCCT). Design and methodologic considerations for the feasibility phase. Diabetes 1986 5;35(5):530–545. 2869996

[11] Early Treatment Diabetic Retinopathy Study Research Group. Photocoagulation treatment of proliferative diabetic retinopathy: the second report of diabetic retinopathy study findings. Ophthalmology 1978 1;85(1):82–106. 345173 10.1016/s0161-6420(78)35693-1

[12] Prineas RJ, Crow RS, Zhang ZM. The Minnesota Code Manual of Electrocardiographic Findings. 2nd ed London: Springer; 2010.

[13] Klein BE, Davis MD, Segal P, Long JA, Harris WA, Haug GA, et al Diabetic retinopathy. Assessment of severity and progression. Ophthalmology 1984 1;91(1):10–17. 6709313 10.1016/s0161-6420(84)34374-3

[14] Carletta J. Assessing agreement on classification tasks: the kappa statistic. Computational Linguistics 1996;22(2):249–254.

[15] White NH, Sun W, Cleary PA, Danis RP, Davis MD, Hainsworth DP, et al Prolonged effect of intensive therapy on the risk of retinopathy complications in patients with type 1 diabetes mellitus: 10 years after the Diabetes Control and Complications Trial. Arch Ophthalmol 2008 12;126(12):1707–1715. 10.1001/archopht.126.12.1707 19064853 PMC2663518

[16] Kemperman FA, Weber JA, Gorgels J, van Zanten AP, Krediet RT, Arisz L. The influence of ketoacids on plasma creatinine assays in diabetic ketoacidosis. J Intern Med 2000 12;248(6):511–517. 11155144 10.1046/j.1365-2796.2000.00768.x

[17] Greenberg N, Roberts WL, Bachmann LM, Wright EC, Dalton RN, Zakowski JJ, et al Specificity characteristics of 7 commercial creatinine measurement procedures by enzymatic and Jaffe method principles. Clin Chem 2012 2;58(2):391–401. 10.1373/clinchem.2011.172288 22166253

[18] de Boer IH, Sun W, Cleary PA, Lachin JM, Molitch ME, Zinman B, et al Longitudinal changes in estimated and measured GFR in type 1 diabetes. J Am Soc Nephrol 2014 4;25(4):810–818. 10.1681/ASN.2013050557 24309189 PMC3968500

[19] Younes N, Cleary PA, Steffes MW, de Boer IH, Molitch ME, Rutledge BN, et al Comparison of urinary albumin-creatinine ratio and albumin excretion rate in the Diabetes Control and Complications Trial/Epidemiology of Diabetes Interventions and Complications study. Clin J Am Soc Nephrol 2010 7;5(7):1235–1242. 10.2215/CJN.07901109 20448066 PMC2893069

[20] Hubbard LD, Sun W, Cleary PA, Danis RP, Hainsworth DP, Peng Q, et al Comparison of digital and film grading of diabetic retinopathy severity in the diabetes control and complications trial/epidemiology of diabetes interventions and complications study. Arch Ophthalmol 2011 6;129(6):718–726. 10.1001/archophthalmol.2011.136 21670338

[21] Hubbard LD, Danis RP, Neider MW, Thayer DW, Wabers HD, White JK, et al Brightness, contrast, and color balance of digital versus film retinal images in the age-related eye disease study 2. Invest Ophthalmol Vis Sci 2008 8;49(8):3269–3282. 10.1167/iovs.07-1267 18421079

[22] Kramer JR, Bayless ML, Lorenzi GM, Ziegler GK, Larkin ME, Lackaye ME, et al Participant characteristics and study features associated with high retention rates in a longitudinal investigation of type 1 diabetes mellitus. Clin Trials 2012 12;9(6):798–805. 10.1177/1740774512458986 23027646 PMC3762976

